# Dynamic volume perfusion CT for preoperative multidimensional resectability assessment of pancreatic ductal adenocarcinoma

**DOI:** 10.3389/fonc.2026.1549875

**Published:** 2026-04-14

**Authors:** Xi Yu, Ting Liu, Jincheng Peng, Qing Zou, Sisi Song, Shiyong Zhang, Xilin Lan, Gang Mai, Lin Yang, Bing Ming

**Affiliations:** 1 Department of Radiology, Deyang People’s Hospital, Deyang, Sichuan, China; 2 Science and Technology Innovation Center, Interventional Medical Center, Department of Radiology, Affiliated Hospital of North Sichuan Medical College, Nanchong, Sichuan, China

**Keywords:** dynamic volume perfusion, pancreatic ductal adenocarcinoma, resectability assessment, tomography, x-ray computers

## Abstract

**Objective:**

The aim of this study was to evaluate the performance of dynamic volume perfusion computed tomography (dVPCT) in the preoperative multidimensional assessment of resectability in patients with pancreatic ductal adenocarcinoma (PDAC).

**Methods:**

We retrospectively collected clinical data from 53 patients with pathologically confirmed PDAC who underwent preoperative epigastric dVPCT. By performing morphological reconstruction, we assessed the degree of involvement of major peripancreatic vessels and compared the predicted resectability status of PDAC lesions against the records from surgical explorations. CT perfusion parameters, including blood flow (BF), blood volume (BV), surface permeability (PS), and mean transit time (MTT), were measured for each patient diagnosed with PDAC. Patients were categorized into resectable and unresectable groups on the basis of the operative findings, and differences in perfusion parameters between these groups were compared via the Mann–Whitney U test. Receiver operating characteristic (ROC) curve analysis was conducted to establish threshold values and areas under the curve (AUCs) for each perfusion parameter as predictors of PDAC resectability.

**Results:**

The sensitivity, specificity, positive prediction value, negative prediction value and accuracy of dVPCT for the diagnosis of peripancreatic arterial invasion were 88.2%, 100%, 100%, 97.8%, and 98.1%, respectively, whereas those for venous invasion were 88.9%, 98.9%, 94.1%, 97.8%, and 97.2%, respectively. For assessing the resectability of PDAC, dVPCT demonstrated a sensitivity of 88.5%, a specificity of 92.6%, a positive predictive value of 92.0%, a negative predictive value of 89.3%, and an accuracy of 90.6%. BF and BV values were significantly lower in the unresectable PDAC group than in the resectable group. The cutoff value for BF for diagnosing resectable PDAC was determined to be 49.9 mL/100 mL/min, with an AUC of 0.732 (95% confidence interval [CI]: 0.593–0.871).Similarly, the cutoff value for BV for the diagnosis of resectable PDAC was 4.3 mL/100 mL, yielding an AUC of 0.714 (95% CI: 0.575–0.853).

**Conclusion:**

Our preliminary study demonstrated that dVPCT permits the simultaneous acquisition of both high-quality morphological images and stable perfusion parameters for PDAC evaluation. These findings suggest that in cases of morphologically uncertain resectability, the derived perfusion parameters may provide complementary information and could serve as adjunctive tools to conventional imaging.

## Introduction

Pancreatic cancer is a highly malignant tumor of the digestive system with a poor prognosis. As demographics have shifted because of the aging population and obesity rates have increased, the incidence of pancreatic cancer has increased alongside the increase in pancreatitis cases. Unfortunately, this increase is accompanied by a dismal prognosis, with only a 13% five-year survival rate. Pancreatic ductal adenocarcinoma (PDAC) is the most prevalent form of pancreatic cancer (1). Currently, negative-margin (R0) tumor resection remains the sole potentially curative treatment approach for PDAC. The application of preoperative neoadjuvant chemotherapy (NCT), either as monotherapy or in combination with radiotherapy (RT), offers an opportunity for surgical intervention in responsive patients (2–4). Accurate preoperative assessment of resectability is crucial for improving patient outcomes; it facilitates informed surgical decisions, minimizes unnecessary invasive procedures, increases R0 resection rates, and reduces postoperative complications (5, 6).

In this era, which is characterized by multidisciplinary collaboration and precise individualized diagnosis and treatment strategies, medical imaging plays a vital role in the preoperative assessment of PDAC resectability. High-quality imaging serves as the foundation for accurate evaluations. One widely utilized framework is provided by the National Comprehensive Cancer Network (NCCN) guidelines for assessing PDAC resectability; these guidelines are based on morphological assessment with multiphase thin-section computed tomography (CT) (2, 3). Multiphase-enhanced thin-section CT is the primary radiographic modality for evaluating PDAC. However, the reliance solely on CT-based morphological assessments presents several limitations. The accuracy of CT in assessing the resectability of PDAC lesions needs improvement; the sPPV (summary positive predictive value) of CT for determining resectability is 81% (7). Moreover, given that PDAC lesions exhibit significant heterogeneity, subjective judgments based exclusively on morphology may compromise individualized therapeutic decision-making (8, 9). Therefore, there is an urgent need for more comprehensive and reliable evidence to assess the resectability of PDAC. Nonmorphological examinations, such as tumor marker detection, genetic testing, and liquid biopsy, play a significant auxiliary role in the early diagnosis and prognostic prediction of PDAC. However, the utility of these indices for assessing PDAC resectability still requires validation through further studies (10–13).

Dynamic volume perfusion CT (dVPCT) is a functional radiological examination that offers a one-stop solution for reconstructing high-quality morphological images. These include enhanced peak images of the arterial phase, venous phase, pancreatic parenchyma phase and delayed phase, as well as vascular–tumor 3D fusion images. Additionally, it performs simultaneous perfusion analysis to obtain functional parameters that reflect blood flow information, thereby providing multidimensional diagnostic insights to assist in clinical decision-making within a single scan (13, 14). Previous research has demonstrated the value of perfusion parameters for the diagnosis, pathological grading and prognostic prediction of PDAC; however, few studies have addressed their application in assessing PDAC resectability (15, 16).

The objective of this study was to explore the clinical value of one-stop dVPCT for assessing the preoperative multidimensional resectability of PDAC.

## Materials and methods

### Patient materials

This study was conducted in accordance with the Declaration of Helsinki and approved by the Ethics Committee of Deyang People’s Hospital (No. 2023-04-096-K01). Imaging and clinical data of patients diagnosed with PDAC who underwent epigastric dVPCT at Deyang People’s Hospital from July 2017 to March 2023 were retrospectively collected. The inclusion criteria were as follows (1): patients with a pathologically confirmed diagnosis of PDAC; (2) completely preserved raw dVPCT scan data and reconstructed images without uncorrectable respiratory motion artifacts; and (3) comprehensive clinical data and intraoperative records containing detailed vascular exploration information. The exclusion criteria were as follows: (1) a time interval between the dVPCT examination and surgery that exceeded 14 days; (2) the coexistence of acute and chronic pancreatitis, diabetes mellitus, or other pancreatic pathologies; (3) concurrent severe cardiac, hepatic, or renal insufficiency; and (4) any patients who had undergone neoadjuvant chemotherapy or chemoradiotherapy before imaging and surgery.

### CT protocol and postprocessing

dVPCT scans were executed via third-generation dual-source CT (Somatom Definition Force; Siemens Healthcare, Germany). Every patient provided signed informed consent prior to the examination. During the examination, a contrast agent (80 ml of iodomelane at 400 mgI/ml) was administered at a rate of 5 ml/s. Advanced iterative reconstruction was performed in DynMulti4D mode. For patients with a BMI less than 25 kg/m², the imaging scanning settings included a tube voltage of 70 kV and a tube current of 100 mA. In cases where the patient’s BMI was above 25 kg/m², the parameters were adjusted to 80 kV tube voltage and 60 mA tube current. The rotation speed was set at 0.3 s, with a z-axis coverage of 17.6 cm. The layer thickness and interval were maintained at 1 mm each. The trigger time was set at 2 s, and the cycle time was 1.5 s. The acquisition protocol consisted of 27 cycles, including 3 sets of 4.5 s, 12 sets of 1.5 s, 3 sets of 3 s, 6 sets of 6.0 s, and 3 sets of 9.0 s, resulting in an overall examination duration of 97.0 s.

The scan data were transferred to a postprocessing workstation (Siemens Healthcare, Germany; Syngo. via) for reconstruction, and the time-attenuation curves (TACs) of the abdominal aorta at the level of the celiac trunk orifice, portal vein trunk, and pancreatic parenchyma were plotted in dynamic angiography mode after applying motion correction and noise reduction techniques. Four nodes surrounding the peak enhancement point were selected and fused into the arterial, pancreatic parenchymal, and portal vein phases, and the terminal four nodes were fused into the delayed phase. In the CT vascular mode, various postprocessing techniques, including multiplanar reconstruction (MPR), curved planar reconstruction (CPR), maximum intensity projection (MIP), and volume rendering (VR), were employed to reconstruct the images of the arterial and portal venous phases for vascular imaging purposes. A region-growth algorithm was used to segment the tumor, which was then displayed on the VR image to generate a 3D vascular–tumor fusion image. The resulting image data were transferred to PACS for further analysis at the workstation.

### Vascular invasion and resectability assessment

The assessment of peripancreatic vascular invasion and tumor resectability status was conducted by two experienced radiologists (Y. X and L. T), each with more than 7 years of experience. They were blinded to the surgical results. Any differences in opinion were resolved through discussion.

The involvement of various vessels, including the celiac artery (CA), common hepatic artery (CHA), gastroduodenal artery (GDA), splenic artery (SA), superior mesenteric artery (SMA), abdominal aorta (AA), portal vein (PV), splenic vein (SV), superior mesenteric vein (SMV) and inferior vena cava (IVC), was graded by the following criteria: Grade 0 indicated no contact between the tumor and blood vessels, with a clear fat gap observed on axial images; Grade 1 represented tumor–vessel contact less than or equal to 180° (abutment) without any abnormality in the vessel contour; and Grade 2 denoted tumor–vessel contact less than or equal to 180° accompanied by abnormality or occlusion in the vascular contour or tumor–vessel contact greater than 180° (encasement). Radiographic resectability assessment was performed for each case of PDAC according to the NCCN Guidelines for Pancreatic Adenocarcinoma (Version 2.2017) (17). As potential clinical goal of our study is to accurately identify a potentially resectable candidate population that may benefit from aggressive multimodal strategies, radiologically resectable and borderline-resectable PDAC were combined into a single category for statistical analysis termed resectable PDAC.

A multidisciplinary team (MDT) discussion was conducted for each patient before surgery. The criteria for surgical exploration of vascular invasion included the presence of a tumor involving blood vessels that are closely adherent to blood vessels or postsurgical pathology indicating invasion of the vascular wall.

### Perfusion analysis

**Figure 1:**
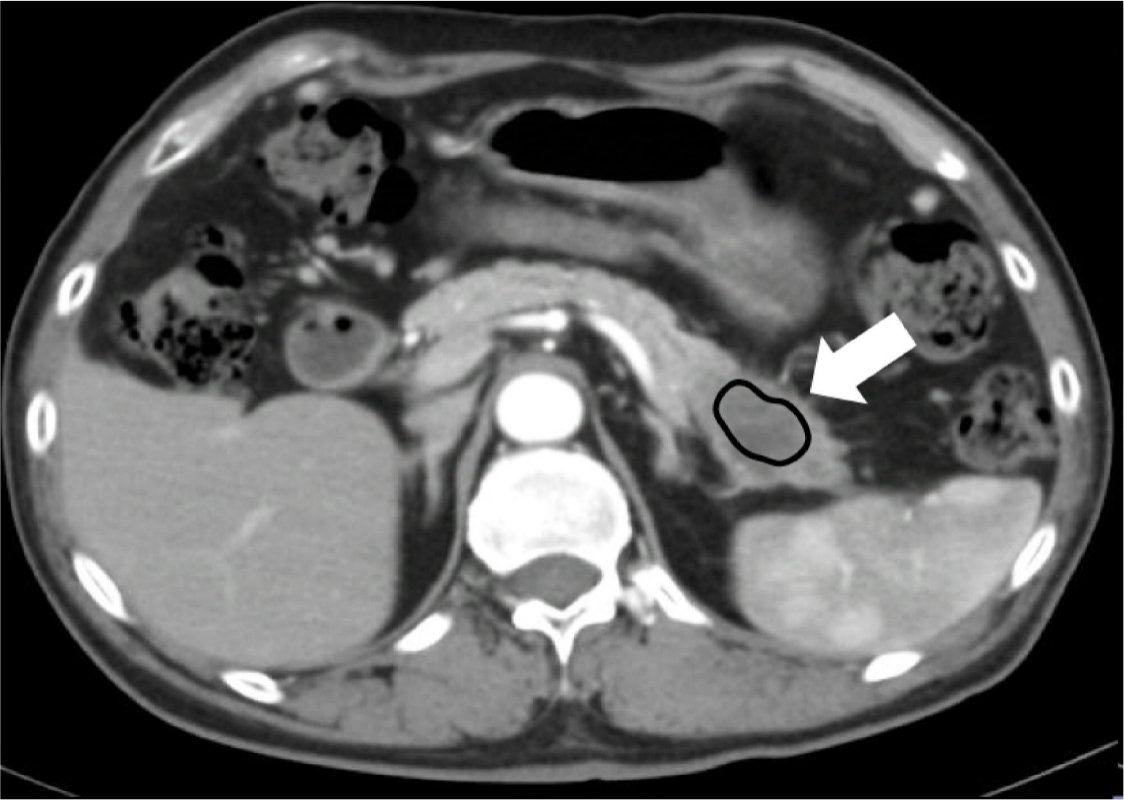
Schematic representation of ROI placement in a patient with PDAC in the pancreatic tail.

A deconvolution algorithm was employed in body perfusion mode to obtain pseudocolor maps of the perfusion parameters after motion correction and noise reduction. The abdominal aorta at the level of the celiac axis orifice was selected as the input artery. The abdominal aorta at the level of the celiac axis orifice was selected as the input artery. Three planes with large axial sections of PDAC were chosen, and three regions of interest (ROIs) with the entire tumor area were manually delineated along the edge of the lesion while avoiding blood vessels, pancreatic ducts, and bile ducts (**Figure 1**). Four parameters, namely, blood flow (BF), blood volume (BV), permeability surface (PS), and mean transit time (MTT), were calculated for these ROIs. The average value from three ROI measurements was considered the final result.

### Radiation dose recording

The volume CT dose index (CTDIvol) and dose length product (DLP) of the dVPCT scans were recorded.

### Statistical analyses

Normally distributed data are presented as the means ± standard deviations (± s), whereas nonnormally distributed data are expressed as median values along with the lower quartile–upper quartile range. The sensitivity, specificity, positive predictive value, negative predictive value, and accuracy of dVPCT for determining arterial and venous invasion as well as for assessing the resectability of PDAC lesions were calculated via a four-grid table method. The Mann–Whitney U test was used to compare differences in PDAC perfusion parameter values between the resectable and unresectable groups. Receiver operating characteristic (ROC) curve analysis was performed to determine the threshold values and area under the curve (AUC) for each perfusion parameter for predicting PDAC resectability.

## Results

### Clinical data and lesion presentation

A total of 53 PDAC patients, comprising 31 males and 22 females, who met these criteria were included in our study. The ages ranged from 47--81 years, with a mean of 64.9 ± 7.6 years. BMI ranged from 15.9--29.6 kg/m^2^, with an average of 24.2 ± 3.5 kg/m^2^. The mean CTDIvol was 31.3 ± 3.0 mGy, and the mean DLP was 646.8 ± 74.6 mGy·cm. Twenty-six patients underwent R0 tumor resection, while 27 underwent exploratory laparotomy with puncture biopsy or other surgical treatments, including 11 cases of biliary-enteric anastomosis, 9 cases of gastric jejunal anastomosis, 2 cases of iodine-125 seed implantation, 2 cases of hyperthermic intraperitoneal chemotherapy, and 1 case of microwave ablation. Postoperative staging revealed 1 case staged as IA, 5 cases as IIA, 11 cases as IB, 10 cases as IIB, 11 cases as III, and 12 cases as IV, along with 3 cases where staging remained undetermined because pathological assessment was not conducted on the lymph nodes. A total of 28 cases exhibited pathological grading: 8 cases (28.6%) demonstrated low–medium differentiation, 16 (57.1%) had medium differentiation, and 4 (14.3%) had high differentiation.

**Figure 2:**
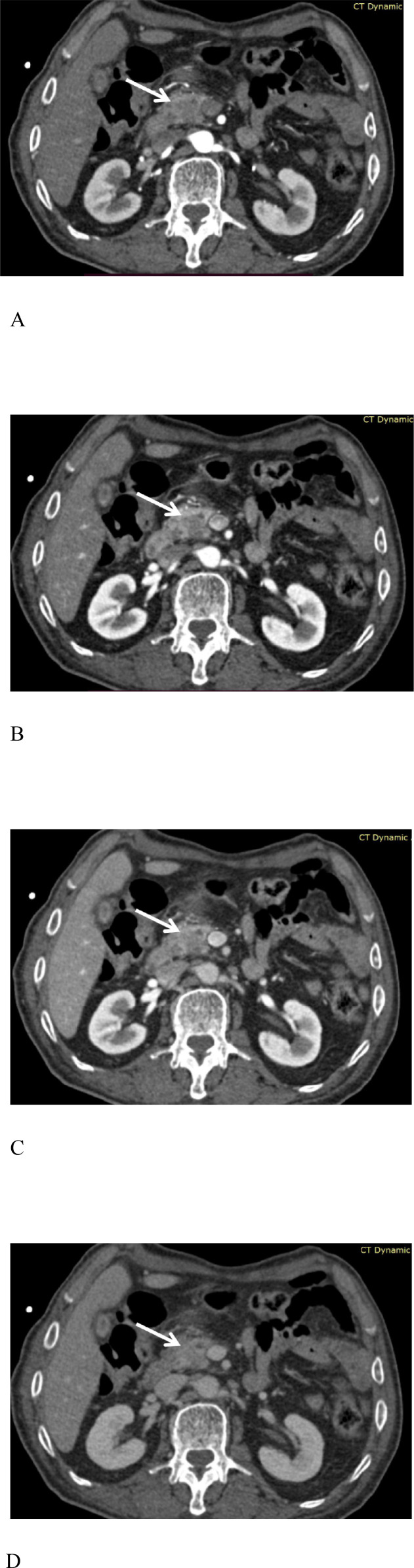
A 66-year-old male patient with stage T2 resectable pancreatic ductal adenocarcinoma (PDAC) (arrow). **(A)** Arterial phase image. The abdominal aorta (AA) and superior mesenteric artery (SMA) reach peak enhancement. A fat plane is visible between the arteries and the tumor, and the tumor contour begins to emerge. **(B)** Pancreatic parenchymal phase image. The contrast agent spreads gradually from the arteries into the pancreas, inducing maximal parenchymal enhancement, and the tumor shows poor enhancement, with the most pronounced contrast being visible to the naked eye compared with that in the other phases, while the arterial enhancement is slightly reduced. **(C)** Venous phase image. The superior mesenteric vein (SMV) exhibits uniform contrast agent filling and has < 180°contact with the tumor. **(D)** Delayed phase image. As the contrast agent is gradually removed, the tumor contour becomes indistinct.

**Figure 3:**
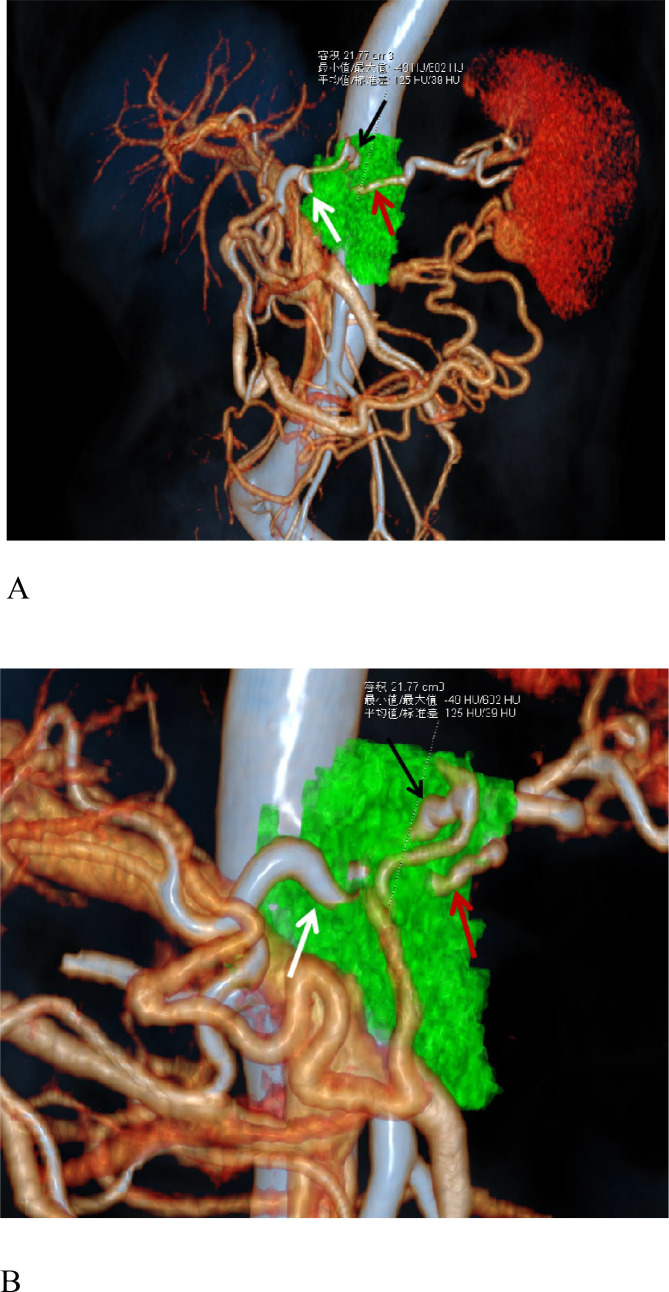
A 67-year-old female patient with ductal adenocarcinoma of the pancreatic body (green) invading the celiac axis and common hepatic artery (white arrow), left gastric artery (black arrow), and splenic artery (red arrow). The image clearly shows the presence of splenic vein–portal venous collaterals. **(A)** Tumor-vessel fusion image. **(B)** Close-up view of the tumor-vessel fusion image.

The lesions were well visualized in all 53 patients with PDAC. Among the PDAC patients, 2 out of 53 showed nearly isointense enhancement in the arterial phase, while 3 patients exhibited isointense enhancement in the portal vein phase. All the tumor foci showed poor enhancement in the pancreatic parenchyma phase, with the most pronounced contrast being visible to the naked eye compared with that in the other phases (**Figure 2**). A homogeneous density was observed in 45 of the PDAC cases, with 8 cases showing cystic necrosis. None of them were accompanied by calcification. In accordance with international guidelines for imaging evaluation of chronic pancreatitis and the literature (18, 19), pancreatic duct dilation is defined as a maximum diameter of the main pancreatic duct > 3 mm. Common bile duct dilation is defined as a maximum diameter > 8 mm (when the gallbladder is present) or > 10 mm (after cholecystectomy). Thirteen patients exhibited the double duct sign, 10 patients had isolated pancreatic duct dilation, and 6 patients had isolated bile duct dilation. Liver perfusion abnormalities were detected in 21 patients. Three-dimensional angiography provides clear visualization of the relationships among the upper abdominal arteries, veins, major organs and lesions in each case, offering practical and effective assistance to ensure smooth operation (**Figure 3**). Sixteen vascular variations within the epigastric region were identified in thirteen patients: 4 alternative right HAs originating from the SMA; 5 alternative left HAs: 2 CA origins, 1 SMA origin, 1 left gastric artery origin, and 1 SA origin; 1 CHA arising from the SMA; 2 hepatic and splenic common trunks of the celiac trunks; 1 gastroduodenal artery branching from the CA, 1 from the left HA; 1 from the left subphrenic artery from the SMA; and 1 SV converging on the left superior mesenteric artery. These vascular variants were accurately identified. The mean peak times and ranges after contrast injection for the abdominal aorta (CA plane), pancreatic parenchyma, and portal vein trunk were as follows: abdominal aorta–mean peak time: 24.7 ± 4.1 s (range: 17.4–29.4 s); pancreatic parenchyma–mean peak time: 31.4 ± 6.5 s (range: 25.9–34.0 s); portal vein trunk–mean peak time: 37.8 ± 2.5 s (range: 32.4–55.0 s).

### Evaluation of vascular invasion

The literature (20) has demonstrated that radiographic grade 2 is utilized for determining vascular invasion, with a higher level of confidence in its accuracy. Among the peripancreatic major vessels involved, the ratios of radiographic invasion to operative invasion were as follows: CA 7/9; CHA 8/8; GDA 6/6; SA 15/16; SMA 9/11; AA 0/1; PV 11/9; SV 12/13; SMV 10/13; IVC 2/2; SV 12/13; SMV 10/13; and IVC 2/2 (**Table 1**). The sensitivity, specificity, positive predictive value, negative predictive value, and accuracy of dVPCT for the determination of arterial invasion were 88.2%, 100%, 100%, 97.8%, and 98.1%, respectively; for venous invasion, the values were 88.9%, 98.9%, 94.1%, 97.8%, and 97.2%, respectively.

**Table 1:** The number of each radiographic grading and intraoperative defined invasion.

Vessel	Radiographic grading			Intraoperative defined invasion
	Grade 0	Grade 1	Grade 2*	
CA	42	4	7	9
CHA	43	2	8	8
GDA	45	2	6	6
SA	35	3	15	16
SMA	36	8	9	11
AA	51	2	0	1
PV	38	4	11	9
SV	38	3	12	13
SMV	36	7	10	13
IVC	50	1	2	2

*Grade 2 indicates radiographic vascular invasion.

CA, celiac artery; CHA, common hepatic artery; GDA, gastroduodenal artery; SA, splenic artery; SMA, superior mesenteric artery; AA, abdominal aorta; PV, portal vein; SV, splenic vein; SMV, superior mesenteric vein; IVC, inferior vena cava.

### Resectability assessment

Among the total patients (53) with PDAC, 26 had resectable tumors, and 27 had unresectable tumors intraoperatively. Among the cases evaluated by dVPCT images, 23 resectable or borderline resectable tumors were correctly identified, whereas 25 unresectable tumors were accurately assessed (**Table 2**). The sensitivity, specificity, positive predictive value, negative predictive value, and accuracy of preoperative resectability based on dVPCT images of patients with PDAC were 88.5%, 92.6%, 92.0%, 89.3%, and 90.6%, respectively.

**Table 2:** Comparison of resectability assessments.

Number	Intraoperative resectable	Intraoperative unresectable	Total
dVPCT resectable/borderline resectable	23	2	25
dVPCT unresectable	3	25	28
Total	26	27	53

dVPCT, dynamic volume perfusion computed tomography.

### Interobserver agreement

Interobserver agreement between vascular invasion and tumor resection probability was determined using a kappa consistency test. The Cohen weighted kappa coefficient for arterial invasion assessment was 0.829 (95% CI: 0.769–0.888); for venous invasion assessment, it was 0.856 (95% CI: 0.792–0.920); and for resectability assessment, it was 0.811 (95% CI: 0.654–0.968), indicating good interobserver agreement.

### Perfusion analysis

**Figure 4:**
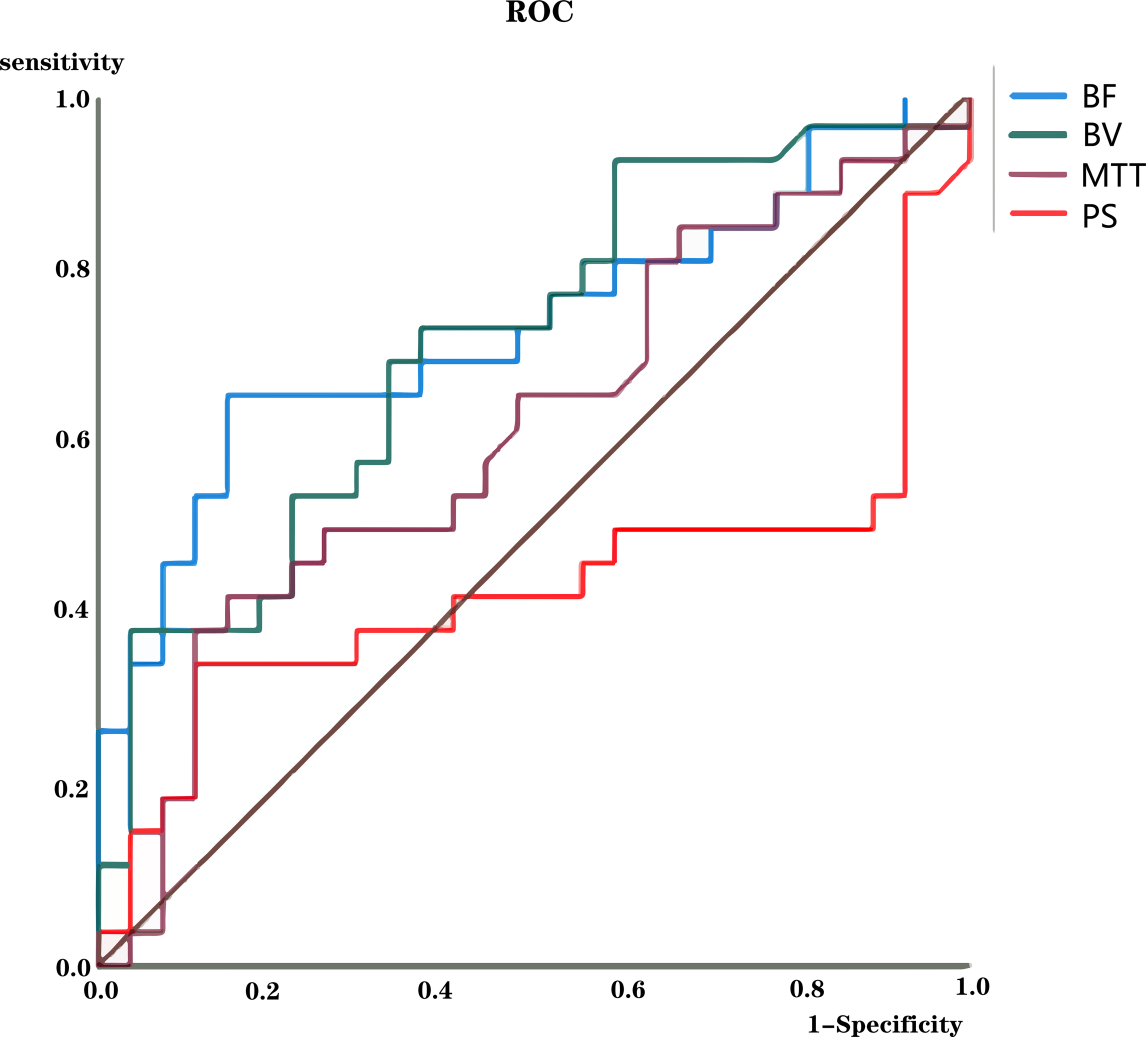
Receiver operating characteristic (ROC) curves for blood flow (BF), blood volume (BV), permeability surface (PS), and mean transit time (MTT) values for assessing the resectability of pancreatic ductal adenocarcinoma.

**Figure 5:**
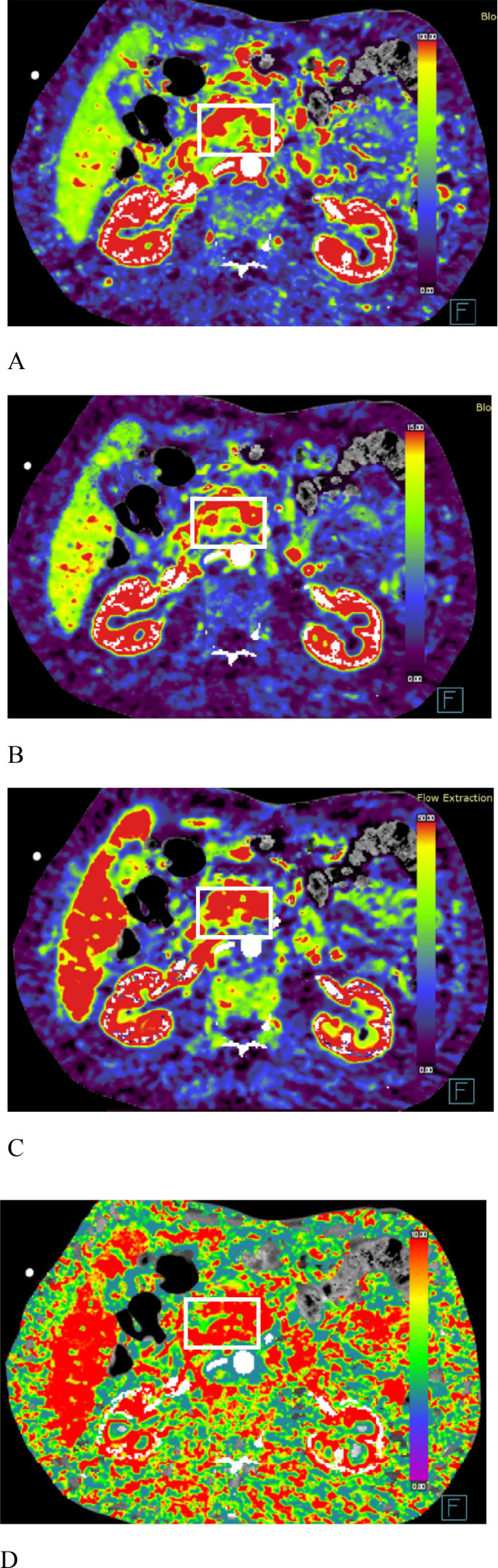
Pseudocolor perfusion images of the BF, BV, PS, and MTT values in patients with PDAC (boxed region) in the same layer are depicted in **Figure 2**. **(A)** BF image. BF = 89.6 mL/100 mL/min (>49.9 mL/100 mL/min); **(B)** BV image. BV = 10.6 mL/100 mL (>4.3 mL/100 mL); **(C)** PS image. PS = 46.3 mL/100 mL/min; **(D)** MTT image. MTT = 8.7 s.

The BF and BV values in patients with PDAC in the unresectable group were significantly lower than those in patients in the resectable group (p<0.05) (**Table 3**). The cutoff BF value for diagnosing resectable PDAC was 49.9 mL/100 mL/min, with an AUC of 0.732 (95% CI: 0.593–0.871). The cutoff BV value for diagnosing resectable PDAC was 4.3 mL/100 mL, with an AUC of 0.714 (95% CI: 0.575–0.853) (**Figure 4**, **Table 4**). There was no significant difference in the PS or MTT values between the two groups. Perfusion pseudocolor maps revealed that perfusion in the PDAC mass was lower than that in the normal pancreatic parenchyma, as demonstrated by decreased BF, BV, and PS values and increased MTT values (**Figure 5**).

**Table 3:** Differences in perfusion parameters between resectable and unresectable groups.

Perfusion parameters	Resectable group	Unresectable group	z value	*p* value
BF	53.1(41.6-62.6)	42.2(36.8-48.9)	-2.900	0.004
BV	5.0(4.1-8.7)	4.1(3.0-5.0)	-2.678	0.007
PS	14.0 (8.7-30.2)	18.1 (14.8-26.3)	-1.423	0.155
MTT	7.0 (6.1-8.8)	6.4(5.6-7.3)	0.810	0.418

Units: BF (blood flow): mL/100 mL/min; BV (blood volume): mL/100 mL; PS (surface permeability): mL/100 mL/min; MTT (mean transit time): s.

**Table 4:** Diagnostic performance of BF and BV values for the assessment of resectability.

Perfusion parameter	Cutoff value	AUC (95% CI)	p value	Sensitivity	Specificity
BF	49.9	0.732(0.593-0.871)	0.004	65%	85%
BV	4.3	0.714(0.575-0.853)	0.007	73%	63%

Units: BF (blood flow): mL/100 mL/min; BV (blood volume): mL/100 mL.

## Discussion

### Assessment of the morphologic resectability of PDAC

The NCCN Guidelines for Pancreatic Adenocarcinoma (2) classify PDAC into three categories, namely, resectable, borderline resectable, and unresectable, on the basis of the association of the tumor with peripheral organs and vessels and the presence of metastases. The assessment of resectability directly influences therapeutic decisions. Morphological evaluation is widely regarded as the most direct and effective assessment method (5, 6). The preferred imaging method for the preoperative diagnosis and staging of PDAC is multiphase-enhanced thin-layer CT. A meta-analysis (21) revealed that the sensitivity and specificity of multiphase enhanced thin-layer CT for determining vascular invasion were 74% and 97%, respectively. The sensitivity and specificity of CT for assessing PDAC resectability (including resectability and borderline resectability) are 81% and 42%, respectively (22). dVPCT outperforms prior multiphase enhanced CT for diagnosing vascular invasion and assessing PDAC resectability, with a relatively acceptable radiation dose (23). The underperformance of traditional scanning may be attributed to the fixed acquisition timing, which fails to account for individual circulatory differences—resulting in nonsignificant PDAC parenchymal intensity contrast or inadequate intravascular contrast filling, thereby reducing the accuracy of tumor size estimation and vascular invasion diagnosis (16, 24). dVPCT is feasible for routine clinical practice, as validated from research to clinical application (14, 23).

Compared with conventional CT, dVPCT can yield a personalized enhancement pattern while obtaining the following: (1) Pancreatic parenchymal phase images. PDACs are tumors with poor blood supply, making the pancreatic parenchymal phase crucial for observing them. Since the peak enhancement time varies among different patients’ pancreatic parenchyma, high-contrast image reconstruction via dVPCT, which is based on each patient’s peak enhancement point, remains stable without being affected by individual circulation differences. A poorly enhanced PDAC mass can be observed in distinct contrast to the surrounding conspicuously enhanced parenchyma, effectively preventing leakage during the diagnosis of “isoenhanced” PDAC and facilitating the evaluation of the tumor and its adjacent tissues. (2) Liver-tumor optimal contrast phase images. Optimal contrast phase images of liver and tumor tissue can be used for the dynamic observation of different enhancement patterns of metastases and liver parenchyma. By reconstructing images at peak times that highlight the most significant disparity between the liver parenchyma and tumor enhancement, this technique enables better visualization of small metastases in the liver and improves detection rates for metastatic foci. (3) Vascular 3D images. Angiography and vascular-tumor 3D fusion images reconstructed by the optimal contrast phase intuitively demonstrate the anatomical relationships among tumors, surrounding organs, and blood vessels. These images help surgeons assess peripancreatic vascular alignment and variations and formulate appropriate surgical strategies. dVPCT provides stable and high-quality images that contribute to the precise assessment of the preoperative morphological resectability of PDAC.

Additionally, CT scans are relatively inaccurate for assessing PDAC resectability after chemotherapy because of difficulties in differentiating fibrosis from tumor infiltration on CT scans (25). However, dVPCT enables the dynamic visualization of tissue enhancement patterns, which aids in tissue differentiation.

In addition, the value of GDA invasion in determining the resectability of PDAC is still uncertain. Recently, Kado et al (26) demonstrated that the postoperative prognosis of patients with GDA involvement is significantly worse than that of patients without GDA involvement. According to the NCCN Guidelines for Pancreatic Adenocarcinoma (version 2.2017) (17), solid tumors contact CA >180° without involvement of the aorta and with intact and uninvolved gastroduodenal arteries, thereby permitting a modified Appleby procedure (borderline resectable); some members prefer this criterion to be in the unresectable category. The resectability assessment of pancreatic cancer does not include SA or SV invasion. However, SA and SV involvement is associated with patient prognosis and influences treatment strategies (27–29). SA and SV invasion was common and well demonstrated by dVPCT.

### Assessment of the nonmorphologic resectability of PDAC

Although morphological evaluation is the most important resectability assessment method, it has limitations owing to its subjectivity. Functional examinations help identify the potential biological behaviors of tumors while providing a more comprehensive, scientific, multidimensional approach for assessing PDAC resectability, accurately guiding clinical decisions (30, 31).

According to the 2017 International Consensus Criteria (ICC), the resectability of PDAC should be assessed on the basis of three factors: anatomy (A), biology (B), and general condition (C) (32). Currently, there are limited reliable nonmorphologic criteria available for assessing the resectability of PDAC. The serum tumor markers CA 19-9 and CEA are typically used in the diagnosis, therapeutic efficacy evaluation, and prognostic assessment of PDAC. However, their value in assessing the resectability of PDAC is controversial, and a standardized cutoff value has not been established. Combining these markers with radiological examinations may improve their usefulness (11, 33). Gerken K et al (13) developed a prognostic model that incorporates CA 19-9 levels, surgical time, and clinical manifestations. The area under the curve (AUC) for assessing PDAC resectability was 0.9 or greater when combined with CT results. This highlights the significant role of combining laboratory examinations with radiological examinations for comprehensive resectability assessment in clinical practice. In the present group, the CA 19-9 levels significantly differed between the resectable and unresectable groups. However, the model based on perfusion parameters combined with CA 19-9 values for predicting PDAC resectability did not improve the efficiency, and the AUCs of the dVPCT and combined models were 0.858 and 0.859, respectively. In the future, it is necessary to increase the number of cases for further verification.

Functional radiology is also commonly employed as a complementary test. PET–CT and PET–MRI assess the biological behavior of the tumor by monitoring the standard uptake value (SUV) of the radiopharmaceutical. These imaging modalities can aid in outcome assessment and recurrence monitoring, whereas whole-body examination facilitates the detection of extrapancreatic metastases (34). Diffusion-weighted imaging using magnetic resonance allows the estimation of tumor-free water diffusion limitations through apparent diffusion coefficient (ADC) values, which reflect tumor cell density. This information can be used to determine the degree of malignancy as well as patient outcome and tumor prognosis (35). Furthermore, genetic testing, biomarker testing for molecular subtyping, and liquid biopsy technology have shown potential value in elucidating the biological behavior of PDAC; however, these methods are still in the research stage (10, 36, 37).

CT perfusion imaging is a form of functional radiography. Perfusion parameters serve as intuitive indicators of tissue blood flow status and have been extensively studied in patients with PDAC (15, 38–42). These studies encompass differential diagnosis, prediction of pathological grading, and assessment of outcomes; some findings are now applicable in routine clinical practice. The origins of the pancreatic head and body tail differ, and whether this affects the hemodynamic perfusion of the normal pancreatic parenchyma and tumors is indeed a point worthy of attention. Xu J et al. (43) explored the perfusion characteristics of normal pancreas tissue. Their results indicated that there was no significant difference between the distribution of BF, BV, and PS values in different regions of the pancreas, mainly because the pancreas has an abundant blood supply with numerous peripheral anastomoses. In the present study, we did not observe differences in the perfusion values of PDAC in different regions. A systematic review (15) comprehensively synthesized existing evidence regarding the perfusion characteristics of PDAC. The BF, BV, and PS values are lower in PDAC tissues than in normal pancreatic parenchyma, whereas the MTT values are greater in PDAC tissues than in normal tissues. This phenomenon can be attributed to the substantial presence of proteoglycans within the tumor stroma of PDAC, which elevates interstitial fluid pressure and compresses the tumor vasculature, leading to reduced blood flow and prolonged peak times. In addition, many stromal cells and immune cells are present in the stromal components. They can reshape the tumor microenvironment of PDAC and mediate immune escape and chemoresistance in PDAC, thereby promoting tumor growth and metastasis (44–46). The mesenchymal tumor phenotype of PDAC is correlated with a histologically poorly differentiated tumor growth pattern (47), resulting in lower BF and BV values for high-grade PDAC than for low-grade variants (15, 39, 40). Zaboriene I et al (41) reported that the MTT parameter value may serve as an effective independent prognosticator for identifying poorly differentiated PDAC. The CT perfusion parameters of the tumor tissue not only display the characteristics of the tumor itself but also may indicate the grade of the tumor. Perhaps G3 tumors could have lower perfusion values and possibly show local spread of the disease more often. Recently, Yang Y et al. (48) reported their initial experience with a modified one-stop, multienergy pancreatic VPCT protocol designed for morphologic analysis and functional perfusion assessment of PDAC. The results revealed that poorly differentiated PDAC patients presented significantly lower BF, BV, TTD, and Tmax values and lower lesion-to-parenchyma ratios of BF, BV, and FEP than moderately differentiated to well-differentiated PDAC patients did, with potential implications for improved clinical management. Therefore, poorly differentiated tumors also exhibit a greater propensity for peripheral vascular invasion and distant metastases, contributing to worse prognoses and reduced opportunities for resection. Consequently, BF and BV values may theoretically serve as indirect indicators for predicting the resectability of PDAC.

The present study is a preliminary exploration of the application of perfusion parameters in the evaluation of PDAC resectability. The results of this study revealed differences in PDAC perfusion parameters between the resectable group and the unresectable group, and the AUCs for the BF value and BV value with respect to their potential ability to assess PDAC resectability were 0.732 and 0.714, respectively, which were not high. Although the BF and BV values significantly differed between the resectable and unresectable groups, their diagnostic performance remained moderate. Thus, perfusion parameters should be regarded as adjunctive tools that complement morphological assessment rather than as decisive factors in determining resectability. Therefore, the resection status still depends mainly on high-quality imaging information. When the results of a morphological evaluation are uncertain, perfusion parameters can provide supportive auxiliary evidence. Moreover, the integration of nonmorphological clinical indices with perfusion parameters could facilitate the development of a more robust clinical prediction model aimed at enhancing the accuracy of the assessment of resectability; however, further studies are necessary to substantiate these findings. The perfusion parameters were significant in this study, but the absolute values of differential perfusion may vary among different research institutions. The cutoff value is applicable only to our unit, and other units should establish their own standards.

dVPCT has received preliminary favorable feedback from clinicians, indicating that it may be feasible for routine clinical practice. However, tedious postprocessing (approximately 30 minutes of additional time) may restrict its extensive application. With the development of technology, this situation will inevitably improve in the future.

Additionally, in most clinical cohorts, PDAC tends to be poorly differentiated. However, in our cohort, the proportion of moderately differentiated tumors was relatively high, possibly because of case selection bias or referral patterns. Crippa S et al. (49) reported a total of 502 patients who underwent resection for PDAC. Their data revealed that well-differentiated (G1), moderately differentiated (G2), and poorly differentiated (G3) PDAC was present in 23 (4.5%), 310 (62%), and 169 (33.5%) patients, respectively. The R0 resection rates decreased as the histologic grade increased from G1 to G2 and to G3. This may be another reason for the high proportion of moderately differentiated tumors in the present group.

### Limitations

This study has the following limitations: (1) This was a single-center retrospective analysis with a limited sample size. In the future, prospective studies with data from multiple centers and large sample sizes are needed for further verification (including external verification). (2) The resectable PDAC group included both resectable and borderline resectable cases, which could not be analyzed in subgroups because of the limited sample size. This methodological choice may influence the results and their interpretation, particularly in relation to clinical decision-making and prognosis. (3) Factors that may influence perfusion differences, such as tumor location and patients’ overall health, were not considered; additionally, the vendor-specific nature of dVPCT and the manual delineation of ROIs may limit reproducibility.

### Conclusion

Our preliminary study demonstrated that dVPCT permits the simultaneous acquisition of both high-quality morphological images and stable perfusion parameters for PDAC evaluation. These findings suggest that in cases of morphologically uncertain resectability, the derived perfusion parameters may provide complementary information and could serve as adjunctive tools to conventional imaging.

## Data Availability

The original contributions presented in the study are included in the article/supplementary material. Further inquiries can be directed to the corresponding authors.
